# Integrated evidence reveals a new subspecies of the genus *Seuratascaris* (Nematoda: Ascaridomorpha), with characterization of the complete mitochondrial genome

**DOI:** 10.1051/parasite/2025008

**Published:** 2025-02-24

**Authors:** Xiao-Hong Gu, Jia-Tong Mu, Hui-Xia Chen, Liang Li

**Affiliations:** 1 Hebei Collaborative Innovation Center for Eco‐Environment; Hebei Key Laboratory of Animal Physiology, Biochemistry and Molecular Biology; College of Life Sciences, Hebei Normal University 050024 Shijiazhuang Hebei Province P.R. China; 2 Ministry of Education Key Laboratory of Molecular and Cellular Biology 050024 Shijiazhuang Hebei Province P.R. China

**Keywords:** Nematoda, Ascaridoidea, Amphibia, Integrated taxonomy, ASAP, Mitogenome

## Abstract

Species of *Seuratascaris* Sprent, 1985 are a rarely reported group of ascaridoid nematodes, parasitising various frogs and toads. In the present study, a new subspecies of *Seuratascaris*, *S. physalis bazhaiensis* n. subsp. was described using integrated taxonomic methods, based on specimens collected from *Odorrana graminea* (Anura: Ranidae) in Guangxi Zhuang Autonomous Region, China. Results of the Assemble Species by Automatic Partitioning (ASAP) and Bayesian inference based on the mitochondrial *cox*1, *cox*2 and *rrnS* data all supported *S. physalis bazhaiensis* representing a distinct taxon from the nominate subspecies *S. physalis physalis*. Supplementary morphometric and genetic data of *S. phy. physalis* are presented based on newly collected material from *Odorrana tiannanensis* (Anura: Ranidae) and *Rhacophorus* sp. (Anura: Rhacophoridae) in Yunnan Province, China. A key to species of *Seuratascaris* is provided. The complete mitochondrial genome of *S. physalis bazhaiensis* was sequenced and annotated, and represents the first mitogenomic data for the genus *Seuratascaris*. This mitogenome has only 13,628 bp (including 12 protein-coding genes, 22 tRNA genes, 2 ribosomal RNAs, and only 1 non-coding region), and is the smallest of the reported ascaridoid mitogenomes so far.

## Abstract (fr)

Les espèces de *Seuratascaris* Sprent, 1985 sont un groupe rarement signalé de nématodes ascaridoïdes, parasitant diverses grenouilles et crapauds. Dans la présente étude, une nouvelle sous-espèce de *Seuratascaris*, *S. physalis bazhaiensis* n. subsp. a été décrite à l’aide de méthodes taxonomiques intégrées, basées sur des spécimens collectés chez *Odorrana graminea* (Anura : Ranidae) dans la région autonome Zhuang du Guangxi, en Chine. Les résultats de l’assemblage d’espèces par partitionnement automatique (ASAP) et de l’inférence bayésienne basée sur les données mitochondriales *cox*1, *cox*2 et *rrnS* ont tous montré que *S. physalis bazhaiensis* représentait un taxon distinct de la sous-espèce nominale *S. physalis physalis*. Des données morphométriques et génétiques supplémentaires pour *S. phy. physalis* sont présentées sur la base de matériel nouvellement collecté à partir d’*Odorrana tiannanensis* (Anura : Ranidae) et de *Rhacophorus* sp. (Anura : Rhacophoridae) dans la province du Yunnan, en Chine. Une clé des espèces de *Seuratascaris* est fournie. Le génome mitochondrial complet de *S. physalis bazhaiensis* a été séquencé et annoté, et représente les premières données mitogénomiques pour le genre *Seuratascaris*. Ce mitogénome ne compte que 13,628 pb (dont 12 gènes codant pour des protéines, 22 gènes d’ARNt, 2 ARN ribosomiques et seulement 1 région non codante), et est le plus petit parmi les mitogénomes des ascaridoïdes rapportés jusqu’à présent.

## Introduction

Species of *Seuratascaris* Sprent, 1985 (Ascaridida: Ascaridoidea) are a rarely reported group of ascaridoid nematodes, specifically parasitizing various frogs and toads [3, 4, 17, 24]. Sprent (1985) [24] erected the genus *Seuratascaris*. Later, Fagerholm (1991) [5] assigned this genus to the Angusticaecinae Skrjabin & Karokhin, 1945 in the Ascarididae Baird, 1853. To date, only four nominal species have been reported from Europe, Oceania and South East Asia, namely *S. numidica* (Seurat, 1917), *S. ranae* (Wang, Zhao & Chen, 1978), *S. physalis* Chen & Li, 2023 and *S. schmackeri* Liu, Fang, Zheng & Wu, 2023 [3, 4, 17, 24, 25]. Although the recent studies sequenced some nuclear and mitochondrial data of this group [3, 4], there has been no mitochondrial genomic data for species of *Seuratascaris* reported.

In the present study, some specimens of *Seuratascaris* collected from *Odorrana graminea* (Boulenger) (Anura: Ranidae) in China, were identified to species level using integrated morphological methods (light and scanning electron microscopy) and molecular approaches [sequencing the nuclear internal transcribed spacer (ITS) regions and mitochondrial cytochrome c oxidase subunit 1 (*cox*1), cytochrome c oxidase subunit 2 (*cox*2) and small subunit ribosomal RNA gene (*rrnS*)]. The Assemble Species by Automatic Partitioning (ASAP) analyses and Bayesian inference (BI) based on the ITS, *cox*1, *cox*2 and *rrnS* sequence data, were also used for species delimitation of *Seuratascaris* spp., respectively. In order to further reveal the characterization of mitochondrial genome of ascaridoid nematodes, the mitogenome of the present material was also sequenced and annotated, which represents the first mitogenomic data for the genus *Seuratascaris*.

## Materials and methods

### Ethics

This study was conducted under the protocol of Hebei Normal University (LLSC2024090). All applicable national and international guidelines for the protection and use of animals were followed.

### Morphological observation

A total of 21 individuals of *Odorrana graminea* were caught by hand at night in Bazhaigou Scenic Area, Qinzhou city, Guangxi Zhuang Autonomous Region, China, and euthanized by the double marrow destruction method [12]. Nematode specimens were collected from the digestive tract. Parasites were washed in physiological saline, then stored in 80% ethanol until studied. The morphology of several specimens of *S. phy. physalis* newly collected from *Odorrana tiannanensis* (Yang & Li) (Anura: Ranidae) and *Rhacophorus* sp. (Anura: Rhacophoridae) in Yunnan Province, China, were also studied.

For light microscopic study, nematodes were cleared in lactophenol. Photomicrographs were recorded using a Nikon^®^ digital camera coupled to a Nikon^®^ optical microscope (Nikon ECLIPSE Ni-U, Nikon Corporation, Tokyo, Japan). For scanning electron microscopy (SEM), the cephalic extremity and posterior end of one male and one female were re-fixed in 4% formaldehyde solution, post-fixed in 1% OsO_4_, dehydrated *via* an ethanol series and acetone, and then critical point dried. Samples were coated with gold and examined using a Hitachi S-4800 scanning electron microscope at an accelerating voltage of 20 kV. Measurements are given in millimetres (mm). Nematode specimens were assigned to the genus *Seuratascaris* based on the following features according to previous studies [3, 4, 24], including lips possessing dentigerous ridges, the absence of interlabia, ventriculus, ventricular appendage and gubernaculum, the excretory pore just posterior to the nerve ring, the presence of an intestinal caecum and spicules very short without alae.

### Molecular procedures

The mid-body of 2 nematode individuals (1 male and 1 female) collected from *O. graminea* in Guangxi, and 3 individuals of *S. phy. physalis* (1 male and 1 female from *Rhacophorus* sp., and 1 female from *O. tiannanensis*) were used for molecular analysis. Genomic DNA was extracted using a Column Genomic DNA Isolation Kit (Shanghai Sangon, Shanghai, China), according to the manufacturer’s instructions. DNA was eluted in elution buffer and kept at −20 °C until use. The used primers and cycling conditions for amplifying the target sequences of ITS, *cox*1, *cox*2 and *rrnS*, and the procedures for sequencing and analysing were according to previous studies [3, 10]. All of the sequences obtained herein were deposited in the GenBank database (http://www.ncbi.nlm.nih.gov) under the following accession numbers: the present new subspecies: ITS: PQ535527, PQ535528; *cox*1: PQ535571, PQ535572; *cox*2: PQ554528, PQ554529; *rrnS*: PQ535525, PQ535526. *S. phy. physalis*: ITS: PQ818925–PQ818927; *cox*1: PQ817697–PQ817699; *cox*2: PQ819747–PQ819749; *rrnS*: PQ818928–PQ818930.

### ASAP and BI analyses

The ASAP analyses were executed using the ASAP online server (https://bioinfo.mnhn.fr/abi/public/asap) under the Kimura (K80) ts/tv model based on the ITS, *cox*1, *cox*2 and *rrnS* sequence data, respectively. The results of ASAP with lowest scores were considered as the optimal group number in the present study. Bayesian inference was performed using MrBayes 3.2.7 with two parallel runs (1,000,000 generations) under the following optimal models (*i.e.*, HKY + G model for *cox*1, HKY + G model for *cox*2, HKY + G model for *rrnS*, and DAYHOFF + I model for ITS). *Ophidascaris baylisi* (Robinson) (Ascaridoidea: Ascarididae) was chosen as the out-group for both ASAP and BI analyses.

### Mitochondrial genome sequencing, assembly and annotation

A total of 35 Gb clean genomic data were generated using the Pair-End 150 sequencing method on the Illumina NovaSeq 6000 platform by Novogene (Tianjin, China). The complete mitochondrial genomes were assembled using GetOrganelle v1.7.2a [14]. Protein coding genes (PCGs), rRNAs and tRNAs were annotated using MitoS web server (http://mitos2.bioinf.uni-leipzig.de/index.py) and MitoZ v3.6 [18]. The open reading frame (ORF) of each PCG was confirmed manually by the web version of ORF finder (https://www.ncbi.nlm.nih.gov/orffinder/). The “lost” tRNA genes ignored by both MitoS and MitoZ, were identified using BLAST based on a database of the existing tRNA sequences of nematodes. The secondary structures of tRNAs were predicted by ViennaRNA module [8], building on MitoS2 [2] and RNAstructure v6.3 [22], followed by manual correction. The base composition, amino acid usage and relative synonymous codon usage (RSCU) were calculated by Python script, which refers to the Codon Adaptation Index (CAI) [15]. The total length of the base composition included ambiguous bases. The base skew analysis was used to describe the base composition of nucleotide sequences. The complete mitochondrial genome of the present material obtained herein was deposited in the GenBank database (http://www.ncbi.nlm.nih.gov) under accession number PQ468471.

## Results

## *Seuratascaris physalis bazhaiensis* n. subsp. (Figs. 1–4)

urn:lsid:zoobank.org:act:9B09A6D3-8E5A-46BB-81EE-70A549A0A951

**Figure 1:**
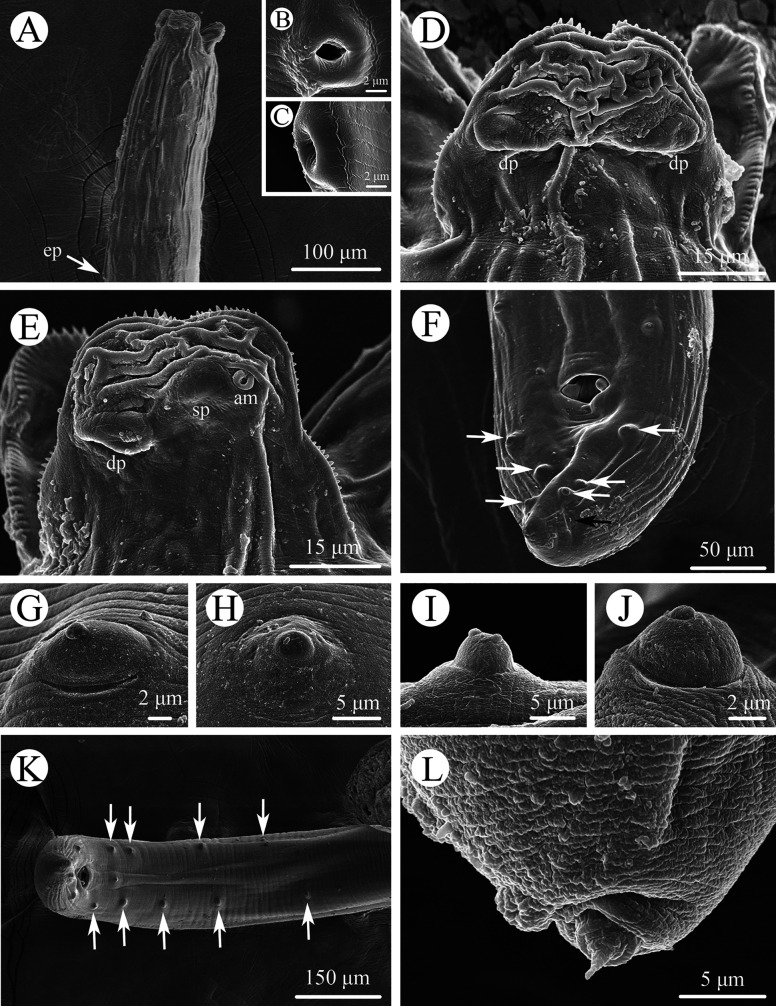
Scanning electron micrographs of *Seuratascaris physalis bazhaiensis* n. subsp. collected from *Odorrana graminea* (Boulenger) (Anura: Ranidae) in China, male. A: anterior part of body (excretory pore arrowed), lateral view; B: magnified image of excretory pore, ventral view; C: magnified image of excretory pore, lateral view; D: dorsal lip; E: subventral lip; F: tail (postcloacal papillae indicated using white arrows, phasmid indicated using black arrow), ventral view; G: magnified image of precloacal papillae; H: magnified image of medio-ventral precloacal papilla; I: magnified image of postcloacal double papillae; J: magnified image of postcloacal papillae; K: posterior end of body (precloacal papillae arrowed), ventral view; L: magnified image of tail tip. *Abbreviations*: am, amphid; dp, double papilla; sp., single papilla.

*Type host*: *Odorrana graminea* (Boulenger, 1899) (Anura: Ranidae).

*Type locality:* Bazhaigou Scenic Area, Guangxi Zhuang Autonomous Region, China.

Site of infection: Intestine.

*Level of infection*: 14.3% (3 out of 21 individuals of *O. graminea*) were infected with intensity of 2.0–4.0 (3.0) nematodes.

*Type specimens*: Holotype: male (HBNU–N–A20240920GL); allotype: female (HBNU–N–A20240921GL); paratypes: 1 male, 4 females (HBNU–N–A20240922GL), deposited in the College of Life Sciences, Hebei Normal University, Hebei Province, China. Paratypes: 2 females (NZMC–PN_506–507), deposited in the National Zoological Museum, Beijing, China.

*Etymology*: The subspecies name refers to the type locality (Bazhaigou Scenic Area).

### Morphology

**Figure 2:**
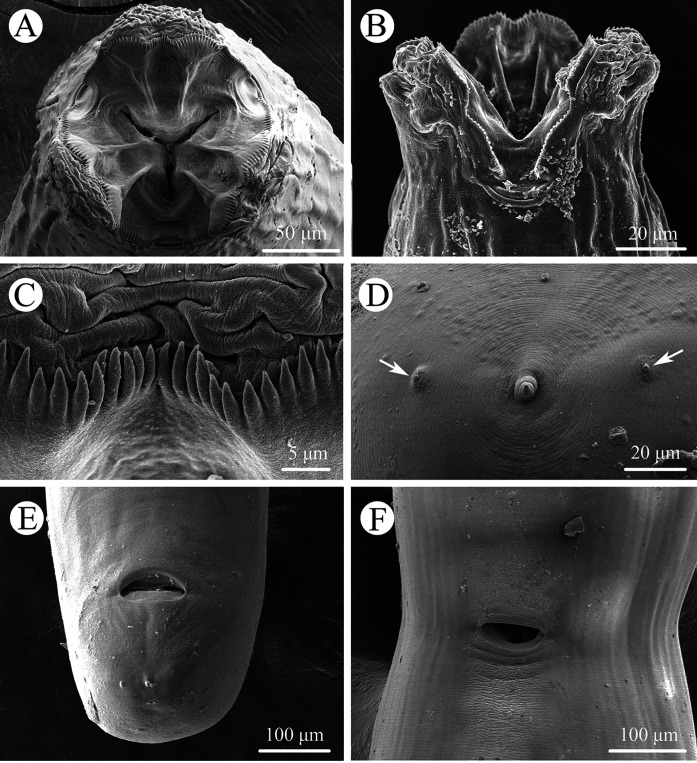
Scanning electron micrographs of *Seuratascaris physalis bazhaiensis* n. subsp. collected from *Odorrana graminea* (Boulenger) (Anura: Ranidae) in China, female. A: cephalic end, apical view; B: cephalic end, lateral view; C: magnified image of denticles; D: magnified image of tail tip (phasmids arrowed); E: posterior end of body, ventral view; F: region of vulva, ventral view.

**Figure 3:**
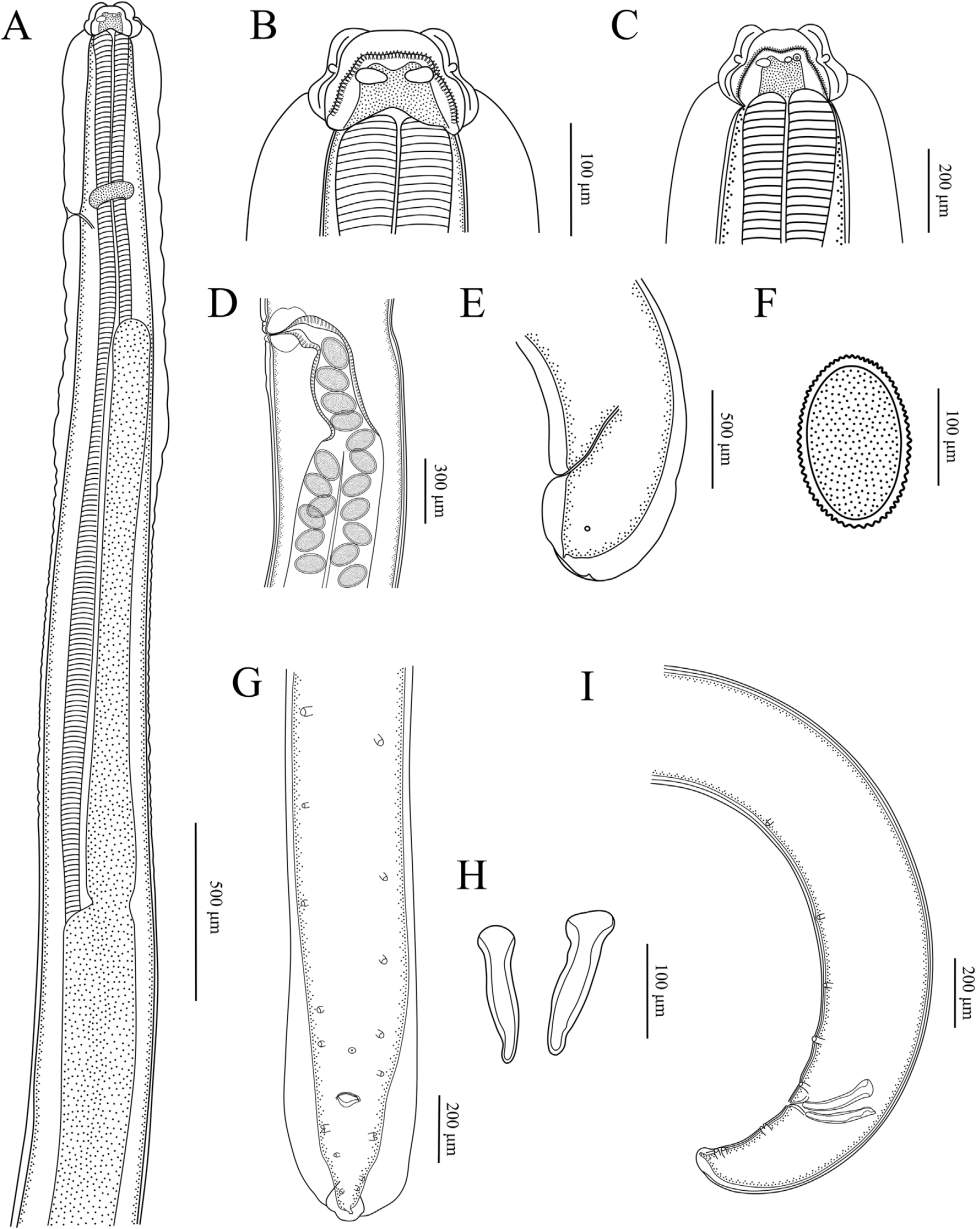
*Seuratascaris physalis bazhaiensis* n. subsp. collected from *Odorrana graminea* (Boulenger) (Anura: Ranidae) in China. A: anterior part of male body, lateral view; B: cephalic end of male, dorsal view; C: cephalic end of male, sublateral view; D: region of vulva, lateral view; E: tail of female, lateral view; F: egg; G: posterior end of male, ventral view; H: spicules; I: posterior end of male, lateral view.

**Figure 4:**
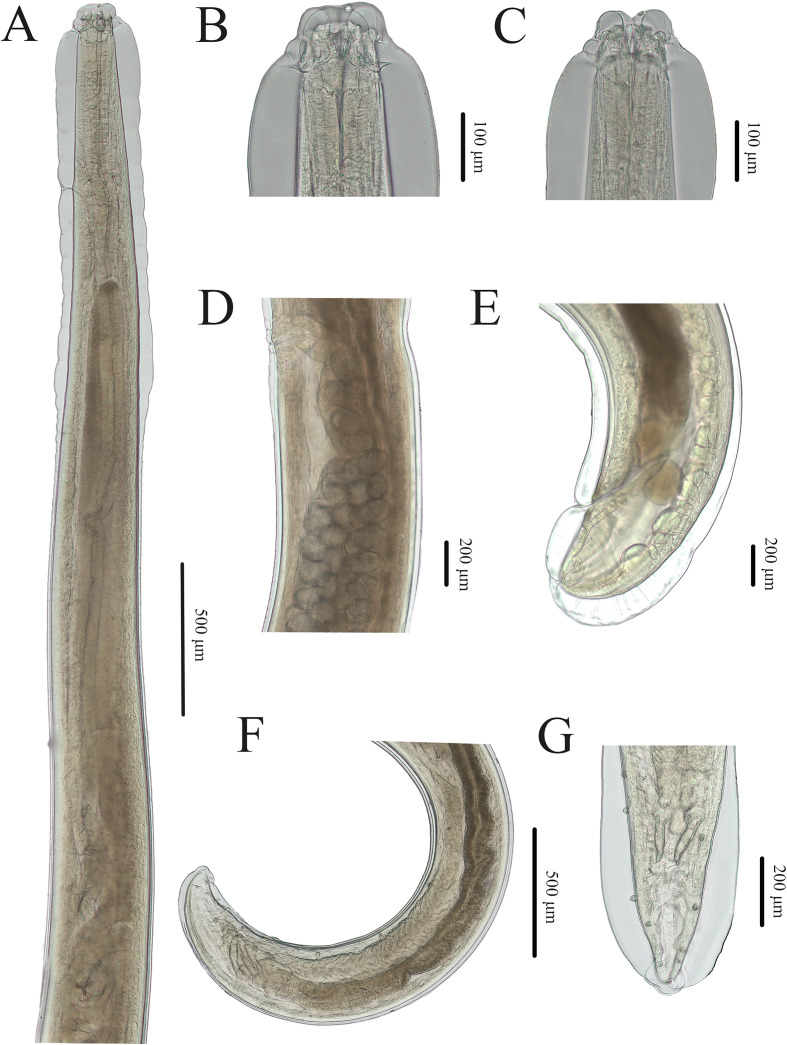
Photomicrographs of *Seuratascaris physalis bazhaiensis* n. subsp. collected from *Odorrana graminea* (Boulenger) (Anura: Ranidae) in China. A: anterior part of male, lateral view; B: cephalic end of male, dorsal view; C: cephalic end of male, ventral view; D: region of vulva, lateral view; E: posterior end of female, lateral view; F: posterior end of male, lateral view; G: posterior end of male, ventral view.

Small to medium sized, whitish nematodes. Cuticle with fine, transverse striations. Maximum width of body at about mid-body. Cephalic extremity with 3 lips, almost equal in size (Figs. 1A, 2A, 2B, 3A–3C, 4A–4C). Dorsal lip with 1 pair of large double cephalic papillae (Figs. 1D, 3B, 4B); subventral lips each with single double cephalic papilla, small papilla and amphid (Figs. 1E, 3C). Distal margin of each lip armed with 75–85 conical denticles and single small triangular, medio-apical notch (Figs. 1D, 1E, 2A–2C). Interlabia absent (Figs. 1A, 2A, 2B, 4C). Cuticle in cervical region inflated to form a cephalic vesicle-like structure extending posteriorly to anterior 1/4 of intestinal caecum (Figs. 3A, 4A). Oesophagus muscular, narrow, nearly cylindrical (Figs. 3A, 4A). Ventriculus and ventricular appendix absent. Intestinal caecum long, about 2/3 of oesophageal length (Figs. 3A, 4A). Nerve-ring at about 1/6 of oesophageal length. Excretory pore just posterior to nerve-ring (Figs. 3A, 4A). Cervical papillae not observed. Cuticle in posterior end of male and female both inflated (Figs. 3E, 3G, 4E, 4G). Tail of both sexes conical, with very small digitiform tip (tail tip retracted, nearly not observed in some individuals under LM) (Figs. 1F, 1K, 1L, 2D, 2E, 3E, 3G, 3I, 4E–4G).

*Male* (Based on 2 mature specimens): Body 10.4–11.5 (10.9) long; maximum width 0.33–0.37 (0.35). Dorsal lip 0.087–0.11 (0.097) long, 0.067 wide. Oesophagus 2.44–2.79 (2.62) mm long, 0.082–0.097 (0.089) in maximum width, representing 23.5–24.3 (23.9)% of body length. Intestinal caecum 1.82–1.84 (1.83) mm long, 0.08–0.11 (0.095) wide, representing 65.9–74.6 (70.3)% of oesophageal length. Nerve-ring 0.42–0.47 (0.45) and excretory pore 0.43–0.47 (0.45) from cephalic extremity, respectively. Posterior end of body distinctly curved ventrally (Figs. 1K, 3I, 4F). Spicules robust, short, without alae, equal in length, blunt at distal end, 0.16–0.17 (0.16) long, representing 1.47–1.49 (1.48)% of body length (Figs. 3H, 3I, 4F, 4G). Gubernaculum absent. Caudal papillae 8–10 pairs in total, arranged as following: 4–6 pairs precloacal (number and/or arrangement of precloacal papillae distinctly asymmetrically on two sides) and 3 pairs postcloacal (first pair being double papillae) (Figs. 1F–1K, 3G, 3I, 4F, 4G). Single medio-ventral precloacal papillae located at some distance anterior to precloacal lip (Figs. 1K, 3G). Precloacal or postcloacal ornamentation absent. Tail 0.25–0.28 (0.27) long. Phasmids small, located laterally, at base of tail tip (Fig. 1F).

*Female* (Based on 7 gravid specimens): Body 27.9–58.2 (46.6) long; maximum width 0.56–1.27 (0.84). Dorsal lip 0.16–0.22 (0.20) long, 0.11–0.18 (0.14) wide. Oesophagus 3.68–7.83 (5.44) long, 0.14–0.20 (0.17) in maximum width, representing 9.92–14.4 (11.8)% of body length. Intestinal caecum 2.59–4.76 (3.16) long, 0.15–0.24 (0.19) wide, representing 49.4–79.5 (58.8)% of oesophageal length. Nerve-ring 0.63–0.92 (0.81) and excretory pore 0.67–0.97 (0.87) from cephalic extremity, respectively. Vulva slit-like, vulval flap absent, 9.41–22.1 (15.6) mm from cephalic extremity, representing 29.7–40.7 (33.4)% of body length (Figs. 2F, 3D, 4D). Vagina muscular, very short; uterus didelphic. Eggs oval, with finely pitted shell, 0.10–0.15 (0.13) × 0.08–0.11 (0.09) in size (*n* = 30) (Figs. 3F, 4D). Tail 0.25–0.44 (0.34) long. Phasmids small, located laterally, at base of tail tip (Figs. 2D, 3E, 4E).

### Remarks

In the genus *Seuratascaris*, *S. phy. bazhaiensis* n. subsp., together with *S. phy. physalis*, can be easily distinguished from *S. ranae* and *S. numidica* by the presence of the distinctly inflated cuticle forming a cephalic vesicle-like structure at the anterior part of the body (*vs.* the absence of an inflated cuticle forming a cephalic vesicle-like structure at the anterior part of the body in the latter two species). Additionally, the new subspecies is distinguished by a relatively longer oesophagus in the male (oesophageal length representing 23.5–24.3% of the body length in *S. phy. bazhaiensis vs* representing about 11.0–20.6% of body length in *S. ranae* and *S. numidica*) [4, 24, 25].

*Seuratascaris physalis physalis* was originally described based only on one male and one female specimen [3]. In the present study, supplementary morphometric data of *S. phy. physalis* were provided based on newly collected specimens from *O. tiannanensis* and *Rhacophorus* sp. in Yunnan Province, China (see Table 1 for details), which can contribute to better understanding of the ranges of intraspecific morphological variation and enable us to diagnose this subspecies more accurately. Chen & Li (2023) [3] reported the cephalic vesicle-like structure extending posteriorly to the level of excretory pore in *S. phy. physalis* based on the limited material. However, in the newly collected specimens of *S. phy. physalis*, we found that the cephalic vesicle-like structure can also extend from the base of the cephalic end nearly to the apex of the intestinal caecum in some individuals. Consequently, in spite of the cephalic vesicle-like structure extending posteriorly to the anterior 1/4 of the intestinal caecum in *S. phy. bazhaiensis*, it is not easy to distinguish this new subspecies from *S. phy. physalis* based on this feature. Although the newly collected male specimens of *S. phy. physalis* also exhibited broad ranges of morphological variability in the lengths of the body and oesophagus, the characteristics of tail length, and the ratio of oesophageal length to body length in the males of *S. phy. physalis* seem to be stable (*S. phy. physalis* with tail 0.10–0.23 mm and oesophageal length representing 14.5–17.9% of body length in males), which are different from that of *S. phy. bazhaiensis* (tail 0.25–0.28 mm and oesophageal length representing 23.5–24.3% of body length in males).

**Table 1:** Morphometric comparisons of *Seuratascaris physalis bazhaiensis* and *Seuratascaris physalis physalis.* Measurements are given in micrometers (μm).

Characteristics	*Seuratascaris physalis bazhaiensis*		*Seuratascaris physalis physalis*		*Seuratascaris physalis physalis*			
	Present study		Chen *et al.* (2018)		Present study		Present study	
	*Odorrana graminea*		*Quasipaa exilispinosa*		*Odorrana tiannanensis*		*Rhacophorus* sp.	
	Male (*n* = 2)	Female (*n* = 7)	Male (*n* = 1)	Female (*n* = 1)	Male (*n* = 1)	Female (*n* = 1)	Male (*n* = 4)	Female (*n* = 5)
BL	10.4–11.5	27.9–58.2	11.3	41.2	8.83	12.5	7.83–32.3	30.5–50.1
OL	2.44–2.79	3.68–7.83	1.63	5.54	1.58	2.35	1.97–3.93	3.62–5.67
OL/BL	23.5–24.3%	9.92–14.4%	14.5%	13.5%	17.9%	18.8%	14.7–16.8%	11.3–12.1%
ICL	1.82–1.84	2.59–4.76	1.11	3.76	0.79	1.06	1.27–1.37	2.00–3.78
ICL/OL	65.9%–74.6%	49.4%–79.5%	67.9%	67.9%	50.0%	45.3%	43.3–64.5%	52.3–70.5%
SL	0.16–0.17	–	0.15	–	0.15	–	0.16–0.23	–
SL/BL	1.47%–1.49%	–	1.32%	–	1.67%	–	0.77–1.15%	–
PRP/PDP/PSP	4–6/0/3	–	6/0/3	–	3/0/3	–	3–5/0/3	–
ES	–	0.10–0.15 × 0.08–0.11	–	0.09–0.14 × 0.08–0.10	–	–	–	0.07–0.13 × 0.07–0.08
TL	0.25–0.28	0.25–0.44	0.18	0.30	0.10	0.17	0.16–0.23	0.10–0.26
VC	–	9.41–22.1	–	14.0	–	3.56	–	12.2–15.5
VC/BL	–	29.7%–40.7%	–	34.0%	–	28.5%	–	27.6–34.8%
Country	China (Guangxi)		China (Yunnan)		China (Yunnan)		China (Yunnan)	

*Abbreviations*: BL – length of body; OL – length of oesophagus; ICL – length of intestinal caecum; SL – length of spicules; NCP – numbers of caudal papillae; ES – size of eggs; TL – length of tail; VC – distance of vulva from cephalic end.

### Key to species/subspecies of Seuratascaris Sprent, 1985

1. Cuticle of the cervical region not inflated to form a cephalic vesicle-like structure...........2.

Cuticle of the cervical region distinctly inflated to form a cephalic vesicle-like structure...........3.

2. Male with 12 pairs of precloacal papillae, vulva located at 56.0% of body length...........***S. ranae****.*

Male with no more than 9 pairs of precloacal papillae, vulva located at 24.7–41.2% of body length...........***S. numidica****.*

3. Tail 0.16–0.23 mm long and oesophageal length representing 14.5–17.9% of body length in male...........***S. physalis physalis****.*

Tail 0.25–0.28 mm long and oesophageal length representing 23.5–24.3% of body length in male...........***S. physalis bazhaiensis* n. subsp**.

### Genetic characterization

#### Partial ITS region

Two ITS sequences of *S. phy. bazhaiensis* obtained herein are both 974 bp in length, with no nucleotide polymorphism detected. In the genus *Seuratascaris*, the ITS sequence data are available in GenBank for *S. numidica* (MG434689, MG434690) and *S. phy. physalis* (OP330325). Pairwise comparison of the ITS sequences of *S. phy. bazhaiensis* with that of *S. numidica* and *S. phy. physalis* available in GenBank displayed 0% (*S. phy. physalis*) to 5.79% (*S. numidica*) nucleotide divergence. Three ITS sequences of *S. phy. physalis* (PQ818925–PQ818927) obtained herein are all 945 bp in length, with no nucleotide polymorphism detected. Pairwise comparison of the newly sequenced ITS data of *S. phy. physalis* with that of *S. phy. bazhaiensis* obtained herein, and *S. phy. physalis* (OP330325) and *S. numidica* (MG434689, MG434690) available in GenBank displayed 0%, 0%, and 5.55% nucleotide divergence, respectively.

#### Partial *cox*1 region

Two *cox*1 sequences of *S. phy. bazhaiensis* obtained herein are both 384 bp in length, with no nucleotide polymorphism detected. In the genus *Seuratascaris*, the *cox*1 sequence data are available in GenBank for *S. numidica* (MG434691, MG434692) and *S. phy. physalis* (OP329215). Pairwise comparison of the *cox*1 sequences of *S. phy. bazhaiensis* with that of *S. numidica* and *S. phy. physalis* available in GenBank displayed 5.73% (*S. phy. physalis*) to 11.2% (*S. numidica*) nucleotide divergence. Three *cox*1 sequences of *S. phy. physalis* (PQ817697–PQ817699) obtained herein are all 384 bp in length, representing two different genotypes, which exhibited 0–0.26% nucleotide divergence. Pairwise comparison of the newly sequenced *cox*1 data of *S. phy. physalis* (PQ817697–PQ817699) with that of *S. phy. bazhaiensis* obtained herein, and *S. phy. physalis* (OP329215) and *S. numidica* (MG434691, MG434692) available in GenBank displayed 5.99–6.25%, 1.82–2.08%, and 12.5–12.8% nucleotide divergence, respectively.

#### Partial *cox*2 region

Two *cox*2 sequences of *S. phy. bazhaiensis* obtained herein are both 501 bp in length, with no nucleotide polymorphism detected. In the genus *Seuratascaris*, the *cox*2 sequence data are available in GenBank for *S. numidica* (OP354281, OP354282) and *S. phy. physalis* (OP354280). Pairwise comparison of the *cox*2 sequences of *S. phy. bazhaiensis* with that of *S. numidica* and *S. phy. physalis* available in GenBank displayed 8.18% (*S. phy. physalis*) to 12.2% (*S. numidica*) nucleotide divergence. Three *cox*2 sequences of *S. phy. physalis* (PQ819747–PQ819749) obtained herein are all 501 bp in length, representing two different genotypes, which exhibited 0–0.60% nucleotide divergence. Pairwise comparison of the newly sequenced *cox*2 data of *S. phy. physalis* (PQ819747–PQ819749) with that of *S. phy. bazhaiensis* obtained herein, and *S. phy. physalis* (OP354280) and *S. numidica* (OP354281, OP354282) available in GenBank displayed 8.18–8.58%, 1.86–2.09%, and 11.1–11.2% nucleotide divergence, respectively.

#### Partial *rrnS* region

Two *rrnS* sequences of *S. phy. bazhaiensis* obtained herein are both 464 bp in length, with no nucleotide polymorphism detected. In the genus *Seuratascaris*, the *rrnS* sequence data are available in GenBank for *S. numidica* (OP331197, OP331198) and *S. phy. physalis* (OP330326). Pairwise comparison of the *rrnS* sequences of *S. phy. bazhaiensis* with that of *S. numidica* and *S. phy. physalis* available in GenBank displayed 4.51% (*S. phy. physalis*) to 8.42% (*S. numidica*) nucleotide divergence. In the present study, we also sequenced the *rrnS* sequences of *S. phy. physalis* based on newly collected specimens from *Rhacophorus* sp. and *O. tiannanensis* in China. Three *rrnS* sequences of *S. phy. physalis* (PQ818928–PQ818930) obtained herein are all 439 bp in length, with no nucleotide polymorphism detected. Pairwise comparison of the newly sequenced *rrnS* data of *S. phy. physalis* (PQ818928–PQ818930) with that of *S. phy. bazhaiensis* obtained herein, and *S. phy. physalis* (OP330326) and *S. numidica* (OP331197, OP331198) available in GenBank displayed 5.01%, 0.91%, and 10.5% nucleotide divergence, respectively.

### ASAP and BI analyses

**Figure 5:**
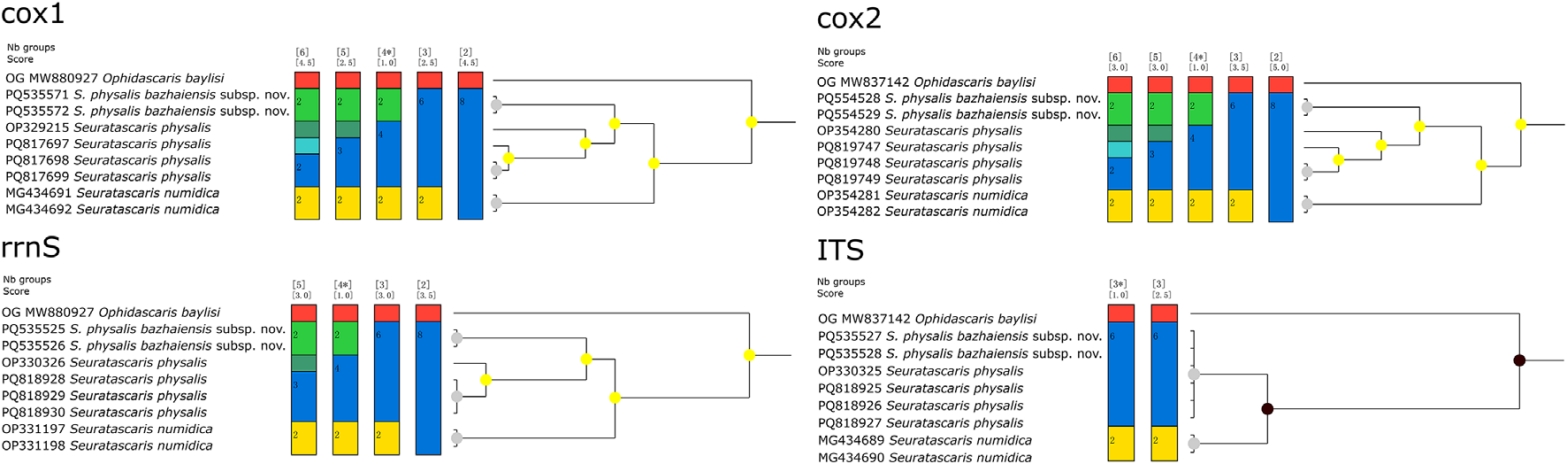
Assemble species by automatic partitioning (ASAP) analyses of *Seuratascaris* spp. based on different nuclear and mitochondrial genetic markers. *Abbreviations*: *cox*1, cytochrome c oxidase subunit I; *cox*2, cytochrome c oxidase subunit II; *rrnS*, Small ribosomal RNA; ITS, internal transcribed spacer; OG, out-group. Asterisk indicated the optimal results recommended by ASAP.

**Figure 6:**
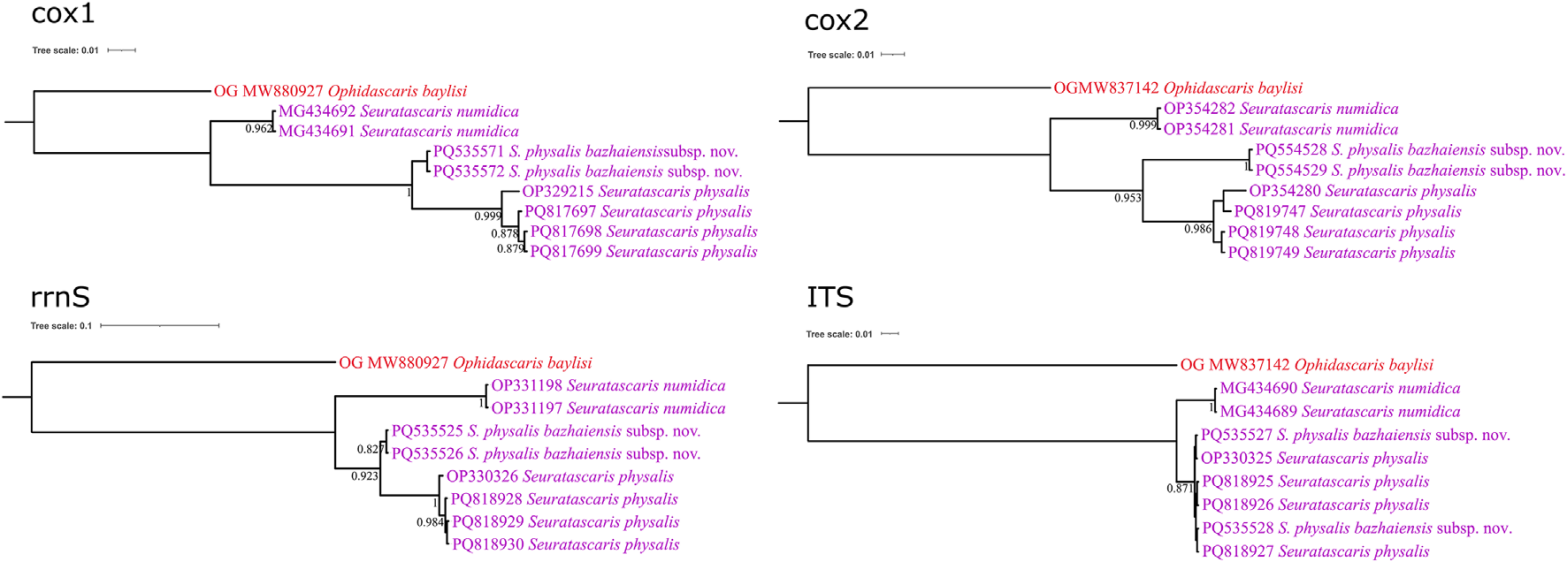
Bayesian inference of *Seuratascaris* spp. based on different nuclear and mitochondrial genetic markers. Bayesian posterior probability values ≥ 0.80 are shown in the phylogenetic trees.

The ASAP and BI results based on the mitochondrial *cox*1, *cox*2, and *rrnS* data all supported the species partition of *S. numidica*, *S. phy. physalis*, and *S. phy. bazhaiensis* n. subsp. However, the present results of ASAP based on the ITS data did not support that *S. phy. physalis* and *S. phy. bazhaiensis* represent two distinct taxa (Fig. 5). The results of analyses using the *cox*1, *cox*2, and *rrnS* data all displayed *S. numidica*, *S. phy. physalis*, and *S. phy. bazhaiensis* n. subsp. as representing three distinct lineages, but the BI results based on the ITS data showed samples of *S. phy. physalis* and *S. phy. bazhaiensis* n. subsp. mixed together (Fig. 6).

### Characterization of the complete mitogenome

**Figure 7:**
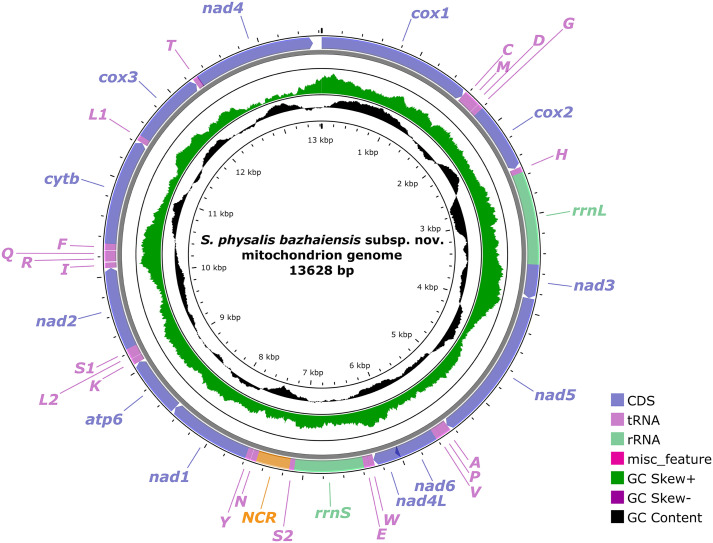
Gene map of the mitochondrial genome of *Seuratascaris physalis bazhaiensis* n. subsp. All 22 tRNA genes are nominated by the one-letter coding with numbers differentiating each of the two tRNAs, serine and leucine.

The circular mitogenome of *S. phy. bazhaiensis* n. subsp. is 13,628 bp in length, and contains 36 genes, including 12 PCGs (missing *atp*8) (*cox*1–3, *cyt*b, *nad*1–6, *nad*4L, and *atp*6), 22 tRNA genes and 2 rRNA genes (*rrnL* located between *tRNA-His* and *nad*3, and *rrnS* located between *tRNA-Glu* and *tRNA-Ser*2) (Fig. 7; Table 2). There is only 1 non-coding region (340 bp) in the mitogenome of *S. phy. bazhaiensis*, located between *tRNA-Ser*2 and *tRNA-Asn*. (Fig. 7; Table 2). All genes are transcribed from the same DNA strand. The overall A + T content in the mitogenome of *S. phy. bazhaiensis* is 73.4%, showing a strong nucleotide compositional bias toward A + T (Table 3). The length of each gene and nucleotide contents of the *S. phy. bazhaiensis* mitogenome are provided (Tables 2 and 3).

**Table 2:** Annotations and gene organization of *Seuratascaris physalis bazhaiensis* n. subsp.

Gene	Type	Start (bp)	End (bp)	Length (bp)	Start Codon	Stop Codon	Anticodon	Strand	Gap or overlap
*cox*1	CDS	1	1575	1575	ATT	TAG		+	−1
tRNA-Cys (C)	tRNA	1575	1630	56			gca	+	0
tRNA-Met (M)	tRNA	1631	1692	62			cau	+	0
tRNA-Asp (D)	tRNA	1693	1748	56			guc	+	2
tRNA-Gly (G)	tRNA	1751	1806	56			ucc	+	0
*cox*2	CDS	1807	2502	696	ATT	TAA		+	4
tRNA-His (H)	tRNA	2507	2562	56			gug	+	0
*rrn*L	rRNA	2563	3508	946				+	0
*nad*3	CDS	3509	3856	348	ATT	TAG		+	2
*nad*5	CDS	3859	5442	1584	ATT	TAG		+	−2
tRNA-Ala (A)	tRNA	5441	5499	59			ugc	+	0
tRNA-Pro (P)	tRNA	5500	5554	55			ugg	+	−1
tRNA-Val (V)	tRNA	5554	5608	55			uac	+	0
*nad*6	CDS	5609	6046	438	TTG	TAA		+	−40
*nad*4L	CDS	6007	6279	273	ATA	TAG		+	0
tRNA-Trp (W)	tRNA	6280	6334	55			uca	+	−1
tRNA-Glu (E)	tRNA	6334	6388	55			uuc	+	0
*rrn*S	rRNA	6389	7092	704				+	0
tRNA-Ser2 (S2)	tRNA	7093	7143	51			uga	+	0
NCR	Non-coding region	7144	7483	340				+	0
tRNA-Asn (N)	tRNA	7484	7540	57			guu	+	2
tRNA-Tyr (Y)	tRNA	7543	7598	56			gua	+	0
*nad*1	CDS	7599	8471	873	TTG	TAG		+	3
*atp*6	CDS	8475	9072	598	TTG	T		+	0
tRNA-Lys (K)	tRNA	9073	9135	63			uuu	+	2
tRNA-Leu2 (L2)	tRNA	9138	9192	55			uaa	+	0
tRNA-Ser1 (S1)	tRNA	9193	9242	50			ucu	+	0
*nad*2	CDS	9243	10085	843	TTG	TAG		+	1
tRNA-Ile (I)	tRNA	10087	10141	55			gau	+	10
tRNA-Arg (R)	tRNA	10152	10205	54			acg	+	0
tRNA-Gln (Q)	tRNA	10206	10259	54			uug	+	5
tRNA-Phe (F)	tRNA	10265	10333	69			gaa	+	−1
*cyt*b	CDS	10333	11425	1093	ATT	T		+	0
tRNA-Leu1 (L1)	tRNA	11426	11482	57			uag	+	0
*cox*3	CDS	11483	12250	768	TTG	TAG		+	2
tRNA-Thr (T)	tRNA	12253	12309	57			ugu	+	−10
*nad*4	CDS	12300	13538	1239	TTG	TAA		+	

**Table 3:** Base composition and skewness of *Seuratascaris physalis bazhaiensis* n. subsp.

Location	A (%)	T (%)	C (%)	G (%)	Total (bp)	A + T (%)	AT skew	GC skew
Whole mitochondrial genome	22.29	51.2	9.76	16.80	13628	73.44	−0.39	0.27
Protein coding genes (PCGs)	19.69	52.50	10.33	17.48	10326	72.19	−0.45	0.26
Condon position								
1st codon	26.28	42.28	10.45	20.99	3442	68.55	−0.23	0.34
2nd codon	18.36	52.15	13.80	15.69	3442	70.51	−0.48	0.06
3rd codon	14.44	63.07	6.74	15.75	3442	77.51	−0.63	0.40
tRNAs	30.57	44.33	8.45	16.65	1243	74.90	−0.18	0.33
rRNAs	29.76	48.06	7.70	14.48	1650	77.82	−0.24	0.31
*rrn*L	28.44	52.01	6.34	13.21	946	80.44	−0.29	0.35
*rrn*S	31.53	42.76	9.52	16.19	704	74.29	−0.15	0.26
NCR	34.41	47.06	8.53	10.00	340	81.47	−0.16	0.08

**Figure 8:**
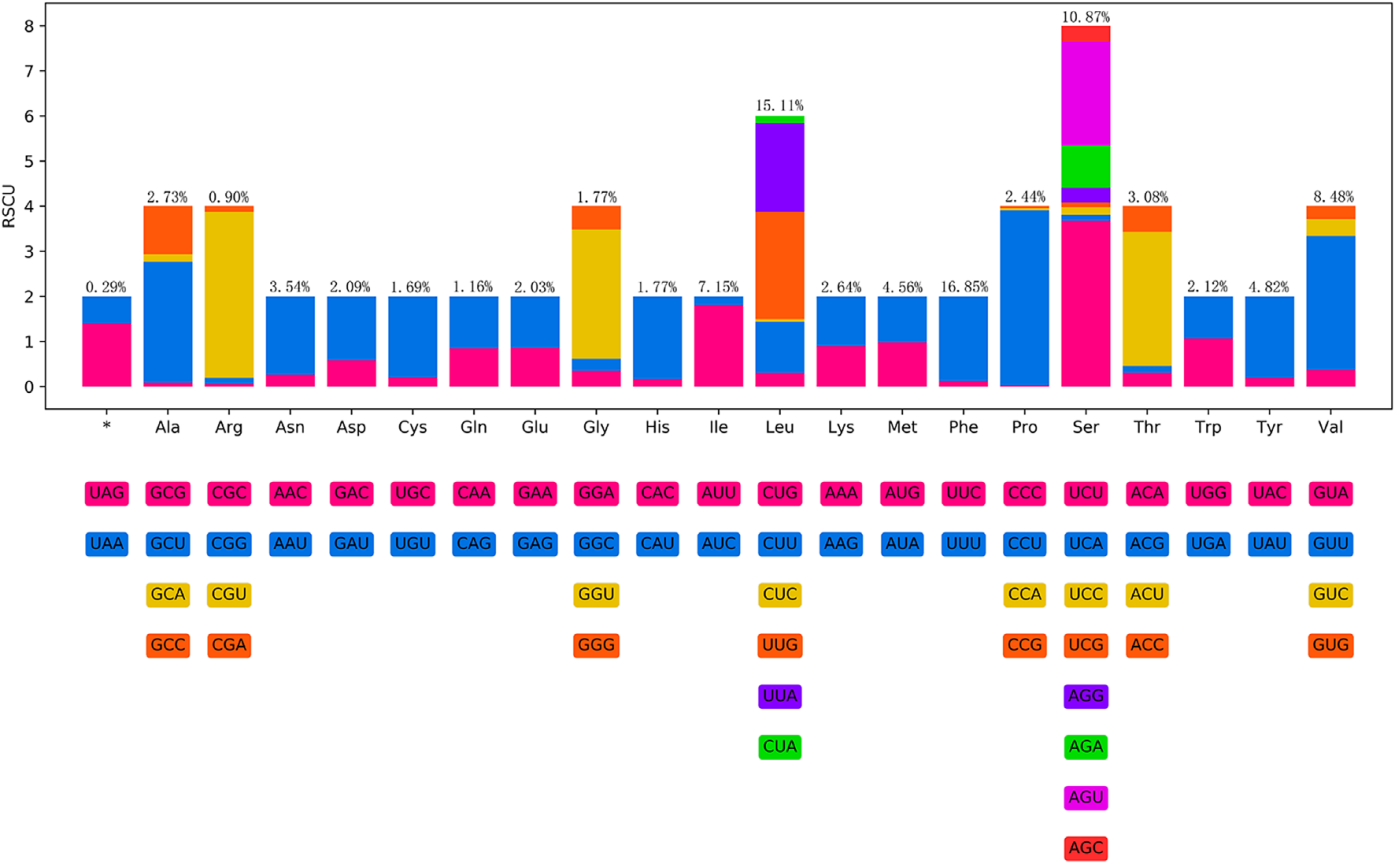
Relative synonymous codon usage (RSCU) of *Seuratascaris physalis bazhaiensis* n. subsp. Codon families (in alphabetical order) indicated below the horizontal axis. Values at the top of each bar represent amino acid usage in percentage.

The total size of the 12 PCGs of *S. phy. bazhaiensis* mitogenome is 10,326 bp (excluding termination codons), ranged in length from 273 bp (*nad*4L) to 1584 bp (*nad*5), which encode 3442 amino acids, respectively (Tables 2 and 3). Among the 12 PCGs, 6 genes (*cox*3, *nad*1–2, *nad*4*, nad*6, and *atp*6) used TTG as the start codon, whereas 5 genes (*cox*1–2, *nad*3, *nad*5, and *cyt*b) used ATT. Only *nad*4*L* used ATA as the start codon. TAG was the most commonly used termination codon for 7 genes, including *cox*1, *cox*3*, nad*1–3, *nad*4L, and *nad*5. Three genes (*cox*2, *nad*4, and *nad*6) used TAA, and the incomplete termination codon T was inferred for *atp*6 and *cyt*b (Table 2). The components and usages of codons in the mitogenome of *S. phy. bazhaiensis* are provided in Figure 8 and in Table 2.

The gene arrangement of 36 genes in the mitogenome of *S. phy. bazhaiensis* belong to the GA3 type for the mitogenome of Nematoda, and is in the following order: *cox*1, *tRNA-Cys*, *tRNA-Met*, *tRNA-Asp*, *tRNA-Gly*, *cox*2, *tRNA-His*, *rrnL*, *nad*3, *nad*5, *tRNA-Ala*, *tRNA-Pro*, *tRNA-Val*, *nad*6, *nad*4L, *tRNA-Trp*, *tRNAGlu*, *rrnS*, *tRNA-Ser*2, *tRNA-Asn*, *tRNA-Tyr*, *nad*1, *atp*6, *tRNA-Lys*, *tRNA-Leu*2, *tRNA-Ser*1, *nad*2, *tRNA-Ile*, *tRNA-Arg*, *tRNA-Gln*, *tRNA-Phe*, *cyt*b, *tRNA-Leu*1, *cox*3, *tRNA-Thr*, *nad*4 (Fig. 7).

## Discussion

Some recent studies proved that it is powerful and practical to utilize the nuclear ITS and mitochondrial *cox*1, *cox*2, and *rrnS* regions as genetic markers for species identification of ascaridoid nematodes [1, 3, 9–11, 19, 23]. The present study revealed the presence of remarkable morphological variability in the lengths of the body, oesophagus, and intestinal caecum, and the number and arrangement of precloacal papillae in *S. phy. physalis* among different individuals collected from different or the same hosts (see Table 1 for details). However, a genetic comparison of the different samples of *S. phy. physalis* collected from three different frog hosts *Quasipaa exilispinosa*, *Odorrana tiannanensis*, and *Rhacophorus* sp., exhibited low levels of nucleotide variation in the ITS (0%), *cox*1 (0–2.08%), *cox*2 (0–2.09%), and *rrnS* regions (0–0.91%), which are distinctly lower than those between *S. phy. physalis* and *S. numidica* (5.55% in ITS, 12.5–12.8% in *cox*1, 11.1–11.2% in *cox*2, and 10.5% in *rrnS*). The present molecular evidence indicated that: (i) the newly collected nematode material from *O. tiannanensis* and *Rhacophorus* sp. in Yunnan Province belongs to *S. phy. physalis*; (ii) the broad ranges of morphological variability in the above-mentioned respects in different individuals of *S. phy. physalis* collected from different frog hosts should be considered as intraspecific variation; (iii) *S. phy. physalis* represents a distinct taxon from *S. numidica*. ASAP and BI analyses based on different nuclear and mitochondrial data supported the present results.

In the present study, we found the presence of differentiable features in the tail length and the ratio of oesophageal length to body length in the male, different geographical distribution areas (Bazhaigou in Guangxi Zhuang Autonomous Region *vs.* Mengla in Yunnan Province), and relatively high level of nucleotide divergence in mitochondrial sequence data (5.73–6.25% in *cox*1, 8.18–8.58% in *cox*2, and 4.51–5.01% in *rrnS*), between the present *Seuratascaris* specimens collected from *O. graminea* in Guangxi and *S. phy. physalis* in Yunan. Additionally, the present ASAP and BI results based on the *cox*1, *cox*2, and *rrnS* sequence data also supported the species partition of these *Seuratascaris* specimens and *S. phy. physalis*. However, we considered that the justification for describing or identifying a new species of zooparasitic nematodes should be based on enough evidence including at least the following four aspects [21], which are the data used to test species boundaries and to delimit the species: (i) presence of morphological characters with taxonomic significance for differentiating the new nominal taxon from its closest related or similar taxa; (ii) presence of ecological differences representing the potential for natural reproductive isolation (*i.e.*, biological, geographical, and host differences); (iii) presence of enough nucleotide variation in some mitochondrial genetic makers (*i.e.*, *cox*1, *cox*2, *rrnS*) representing matrilineal divergence; and (iv) presence of enough nucleotide variation in some nuclear genetic makers (*i.e.*, ITS, 28S) representing patrilineal divergence. Consequently, it seems not clearly evident to erect a new species for our *Seuratascaris* specimens collected from *O. graminea* in Guangxi, because there is no nucleotide divergence in nuclear ITS data detected between the present material and *S. phy. physalis*. However, we prefer to propose a new subspecies *S. phy. bazhaiensis* for the present *Seuratascaris* material collected from *O. graminea* in Guangxi, which can be discriminated from the *S. phy. physalis* based on the differences in morphology, genetics (mtDNA), and geography. *Seuratascaris physalis bazhaiensis* and *S. phy. physalis* may represent “species on the road to differentiation”.

Liu *et al.* (2023) [17] reported *S. schmackeri* Liu, Fang, Zheng & Wu, 2023 from *Odorrana schmackeri* Boettger (Anura: Ranidae) in China, and also provided the ITS (MT434777) and *cox*1 (MN120313) sequence data for *S. schmackeri*. However, the description of *S. schmackeri* is rather poor and, in some cases, the actual generic features are not clear, and some morphometrics are almost certainly erroneous. Additionally, molecular analysis of the genetic data displayed about 98.6% similarity in the ITS region between *S. schmackeri* and *Megalobatrachonema hainanensis* (Cosmocercoidea: Kathlaniidae) (MH545567, MH545568). Furthermore, according to Liu *et al.* (2023) [17], the presence of over 50.0% nucleotide divergence in the *cox*1 sequence between *S. schmackeri* and *S. numidica* also indicated that *S. schmackeri* is not a member of the genus *Seuratascaris*. We made a request to borrow the type material of *S. schmackeri* deposited in Wanna Medical College, China, but failed. Consequently, in order to avoid confusion of the taxonomy of *Seuratascaris*, *S. schmackeri* should be considered as *incertae sedis* or *species inquirenda*.

The current mitogenomic database for ascaridoid nematodes remains insufficient. To date, a total of 33 ascaridoid species with their mitogenomic data are available in GenBank or other databases, but there are no mitochondrial genomic data of *Seuratascaris* species reported so far. The mitogenomes of ascaridoid nematodes seem to exhibit a high degree of conservation in the composition and gene arrangement order. The composition [including 12 PCGs (missing *atp*8), 22 tRNA genes, and 2 rRNA genes] and gene arrangement type (GA3 type) of *S. phy. bazhaiensis* mitogenome agreed well with those of the reported mitogenomes of ascaridoid nematodes [6, 7, 10, 13, 16, 20, 26–31], but *S. phy. bazhaiensis* has only one non-coding region in the mitogenome, which is different from all of the reported ascaridoid mitogenomes, except *Ortleppascaris sinensis* [30]. The size of the complete mitogenome of *S. phy. bazhaiensis* (13,628 bp) represents the smallest mitogenome in the reported ascaridoid mitogenomes so far (13,828–15,045 bp). The mitogenome of *S. phy. bazhaiensis* displayed a strong nucleotide compositional bias toward A + T (73.4%), which is higher than all of the reported mitogenomes of ascaridoid nematodes, except *O. sinensis* (74.0%) [30]. The present study enriched the mitogenomic data and revealed the pattern of mitogenomic evolution of ascaridoid nematodes.

## Conclusion

*Seuratascaris physalis bazhaiensis* n. subsp. was described based on integrated evidence. ASAP and BI results based on the mitochondrial *cox*1, *cox*2 and *rrnS* data all supported *S. phy. bazhaiensis* representing a distinct taxon from *S. phy. physalis*. The supplementary morphometric and genetic data of *S. phy. physalis* based on newly collected specimens from *O. tiannanensis* and *Rhacophorus* sp. would enable us to diagnose this subspecies more accurately. A key to species of *Seuratascaris* is provided. The complete mitochondrial genome of *S. phy. bazhaiensis* was also sequenced and annotated, which represents the first mitogenomic data for the genus *Seuratascaris*. The mitogenome of *S. phy. bazhaiensis* has only 13,628 bp (including 12 protein-coding genes, 22 tRNA genes, 2 ribosomal RNAs, and only 1 non-coding region), and represents the smallest mitogenome of the reported ascaridoid mitogenomes so far.
